# Influence of the Dynamically Disordered N-Terminal Tail Domain on the Amyloid Core Structure of Human Y145Stop Prion Protein Fibrils

**DOI:** 10.3389/fmolb.2022.841790

**Published:** 2022-02-14

**Authors:** Zhe Qi, Krystyna Surewicz, Witold K. Surewicz, Christopher P. Jaroniec

**Affiliations:** ^1^ Department of Chemistry and Biochemistry, The Ohio State University, Columbus, OH, United States; ^2^ Department of Physiology and Biophysics, Case Western Reserve University, Cleveland, OH, United States

**Keywords:** amyloid, prion, octarepeat, intrinsically disordered region/protein, magic angle spinning (MAS) solid-state NMR

## Abstract

The Y145Stop mutant of human prion protein (huPrP23-144) is associated with a familial prionopathy and provides a convenient *in vitro* model for investigating amyloid strains and cross-seeding barriers. huPrP23-144 fibrils feature a compact and relatively rigid parallel in-register *β*-sheet amyloid core spanning ∼30 C-terminal amino acid residues (∼112–141) and a large ∼90-residue dynamically disordered N-terminal tail domain. Here, we systematically evaluate the influence of this dynamic domain on the structure adopted by the huPrP23-144 amyloid core region, by investigating using magic-angle spinning solid-state nuclear magnetic resonance (NMR) spectroscopy a series of fibril samples formed by huPrP23-144 variants corresponding to deletions of large segments of the N-terminal tail. We find that deletion of the bulk of the N-terminal tail, up to residue 98, yields amyloid fibrils with native-like huPrP23-144 core structure. Interestingly, deletion of additional flexible residues in the stretch 99–106 located outside of the amyloid core yields shorter heterogenous fibrils with fingerprint NMR spectra that are clearly distinct from those for full-length huPrP23-144, suggestive of the onset of perturbations to the native structure and degree of molecular ordering for the core residues. For the deletion variant missing residues 99–106 we show that native huPrP23-144 core structure can be “restored” by seeding the fibril growth with preformed full-length huPrP23-144 fibrils.

## Introduction

Most peptide and protein molecules are capable of undergoing conformational conversion from their native state into highly ordered, *β*-sheet rich amyloid fibrils (Dobson, 1999), and for ∼50 human proteins such misfolding and amyloid formation can occur under physiological conditions *in vivo* leading to development of disease (Chiti and Dobson, 2006). A number of amyloids have been found to contain large dynamically disordered domains flanking the structured fibril core (Heise et al., 2005; Siemer et al., 2006; Loquet et al., 2009; Helmus et al., 2010; Bibow et al., 2011; Li et al., 2012; Raveendra et al., 2013; Frederick et al., 2014; Isas et al., 2015; Cervantes et al., 2016; Lin et al., 2017; Murray et al., 2017; Caulkins et al., 2018; Dregni et al., 2019; Fonda et al., 2021), and it has been suggested that the presence of these conformationally flexible domains may be of pathological or functional significance by stabilizing fibril structures and mediating interactions involving protofilaments (Uversky and Fink, 2004; Chiti and Dobson, 2006; Tompa, 2009; van der Wel, 2017; Siemer, 2020). The detailed characterization of dynamically disordered regions in amyloids (and in other large biomacromolecular assemblies) has generally been pursued by multidimensional magic-angle spinning (MAS) nuclear magnetic resonance (NMR) techniques, which are able to visualize these domains directly in hydrated samples at ambient temperature by using experiments based on scalar coupling mediated polarization transfers (Tycko, 2006; van der Wel, 2017; Jaroniec, 2019; Siemer, 2020).

The C-terminally truncated Y145Stop prion protein (PrP23-144) variant is associated with a hereditary prionopathy in humans (Ghetti et al., 1996), and mouse PrP23-144 amyloid fibrils have recently been shown to cause transmissible prion disease in mice (Choi et al., 2016). Importantly, the highly homologous human (hu), mouse (mo) and Syrian hamster (Sha) PrP23-144 proteins (pairwise amino acid, aa, sequence identities of ∼90–95%) have also been shown to provide a valuable *in vitro* model for detailed investigation of the structural basis of amyloid strains and transmissibility barriers (Kundu et al., 2003; Vanik et al., 2004; Jones and Surewicz, 2005; Surewicz et al., 2006). Our previous structural and dynamic solid-state NMR studies of huPrP23-144 fibrils revealed the presence of a structured ∼30-residue parallel in-register *β*-amyloid core (aa ∼112–141) exhibiting limited protein backbone motions on the ∼0.1–1 ms time scale located near the C-terminus and a large dynamically disordered ∼90-residue N-terminal tail domain (aa ∼23–110) (Helmus et al., 2008; Helmus et al., 2010; Helmus et al., 2011; Theint et al., 2018; Aucoin et al., 2019; Shannon et al., 2019). Additional studies of PrP23-144 amyloids containing mutations and deletions corresponding to different huPrP23-144 core residues enabled these sequence modifications to be correlated with structural and dynamic changes in the PrP23-144 amyloid core and provided initial insights into mammalian PrP23-144 cross-seeding specificities (Jones et al., 2011; Theint et al., 2017a; Theint et al., 2017b; Dao et al., 2021).

Previous studies of amyloids formed by full-length prion protein (PrP23-231) and the proteinase-K resistant 90–231 fragment of transmissible spongiform encephalopathy associated mammalian PrP deposits (Prusiner, 1998) suggest that the flexible N-terminal domain may play a role in PrP aggregation properties, and prion structure and pathogenesis (Weissmann, 1999; Lawson et al., 2004; Baskakov and Bocharova, 2005; Frankenfield et al., 2005). The present study aims to assess the influence of the dynamic huPrP23-144 N-terminal region on amyloid assembly and resulting *β*-core conformation. This is achieved by performing systematic solid-state NMR, atomic force microscopy (AFM) and thioflavin T (ThT) fluorescence studies on fibril samples formed *in vitro* from recombinant huPrP23-144 variants corresponding to deletions of large segments of the N-terminal tail. Overall, we find that the majority of dynamically disordered N-terminal tail residues, including the octarepeat region (aa 51–91) implicated in copper binding and homeostasis (Millhauser, 2007; Aguzzi et al., 2008), have little impact on the fibril assembly kinetics and ability of the deletion variants to adopt the native huPrP23-144 amyloid core structure. However, we also find that a stretch of ∼10 conformationally flexible residues that precede the amyloid core region in huPrP23-144 fibrils appears to play a role in the ability to adopt the native core structure and the degree of molecular ordering within the core.

## Results

Previous solid-state NMR studies indicate that the relatively rigid *β*-core region of huPrP23-144 fibrils consists of residues 112–141 (Helmus et al., 2008; Helmus et al, 2010; Helmus et al, 2011; Shannon et al., 2019). In contrast residues 23–111 and 142–144 are not observable in conventional cross-polarization magic angle spinning (CP-MAS) solid-state NMR spectra that utilize dipolar coupling-based polarization transfers, consistent with their increased mobility (van der Wel, 2017; Siemer, 2020) while most of these residues can be detected in MAS NMR spectra utilizing polarization transfers mediated via J-couplings (van der Wel, 2017; Siemer, 2020). To assess the potential influence of the dynamically disordered N-terminal domain of huPrP23-144 on the conformation adopted by the amyloid core region we generated a series of fibril samples from large N-terminal domain deletion variants of huPrP23-144 and examined their fibrillization kinetics, morphologies and molecular conformations by using ThT fluorescence, AFM and solid-state NMR, respectively.

The huPrP23-144 deletion variants employed in these studies spanned residues 28–106—note that the short segment (aa 23–27) containing multiple lysine and arginine residues (as well as the N-terminal GDSP extension present in our huPrP23-144 construct (Helmus et al., 2008) was not deleted in order to ensure the solubility of the different deletion variants. Initially, we investigated the following huPrP23-144 variants: Δ28-50, Δ51-91 (corresponding to deletion of the entire octarepeat region) and Δ92-106 (see Figure 3A for the huPrP23-144 protein sequence and summary of the deletion variants studied). Briefly, fibrils generated from the Δ28-50 and Δ51-91 constructs were found to exhibit wild-type (WT) like morphologies and molecular conformations, while significant differences relative to WT were observed for the Δ92-106 fibrils. To investigate this further we prepared the Δ92-98 variant, which was found to form WT-like fibrils suggesting that deletion of huPrP23-144 N-terminal residues up to aa 98 does not have a significant impact on formation of the native huPrP23-144 amyloid core structure. Based on these findings we then generated the Δ28-98 variant corresponding to the deletion of nearly the entire huPrP23-144 N-terminal tail. While the Δ28-98 construct expressed at reasonable level in rich medium it was found not to express at sufficiently high level in ^13^C and ^15^N isotope enriched minimal medium to permit multidimensional solid-state NMR studies, which led us to generate an additional, Δ28-91, deletion variant. The studies of all the aforementioned huPrP23-144 N-terminal deletion variants are described in additional detail below.

**FIGURE 1:**
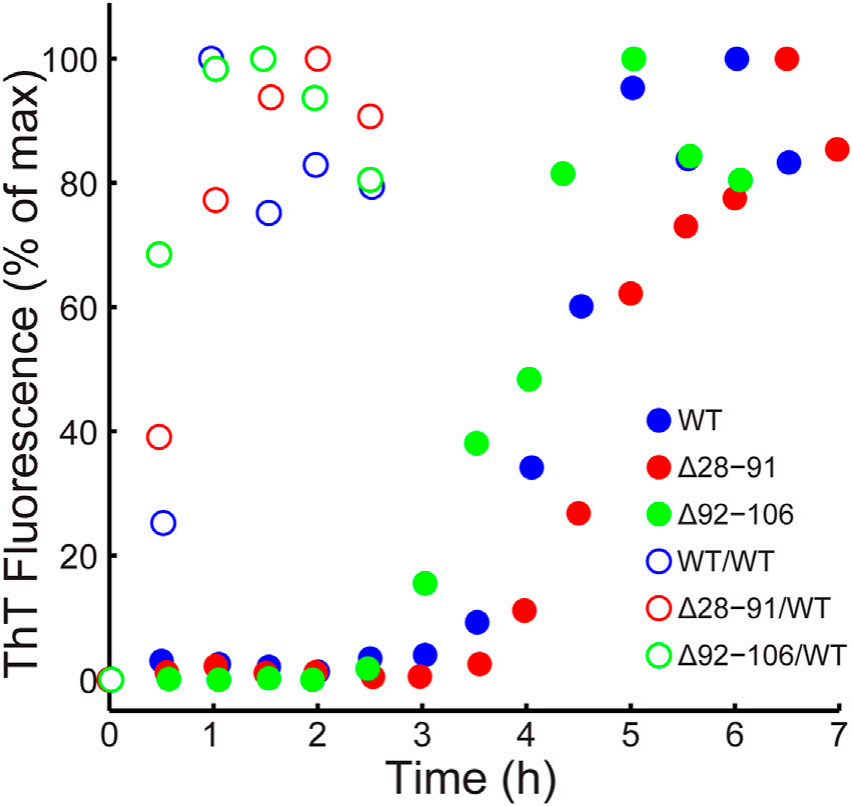
Kinetics of amyloid formation monitored by thioflavin T fluorescence in the absence (filled circles) and presence (open circles) of 2% (mole fraction) of WT huPrP23-144 fibril seeds for WT (blue), Δ28-91 (red) and Δ92-106 (green) huPrP23-144.

As noted above, all the huPrP23-144 deletion variants investigated in this study (Δ28-50, Δ28-91, Δ28-98, Δ51-91, Δ92-98 and Δ92-106) readily converted to amyloid fibrils in autocatalytic, unseeded reactions carried out in potassium phosphate buffer at pH 6.4. The kinetics of fibril formation were monitored by the standard ThT binding assay (Naiki et al., 1989) revealing a nearly identical ∼3-4 h lag phase for WT huPrP23-144 and all the deletion variants at 400 μM protein concentration, in line with the value reported previously for WT huPrP23-144 (Kundu et al., 2003). Representative data for WT, Δ28-91 and Δ92-106 huPrP23-144 are shown in Figure 1. Furthermore, we found that addition of a small amount of pre-formed WT huPrP23-144 fibril seeds to the reaction resulted in complete elimination of the lag phase for all deletion variants studied (Figure 1).

**FIGURE 2:**
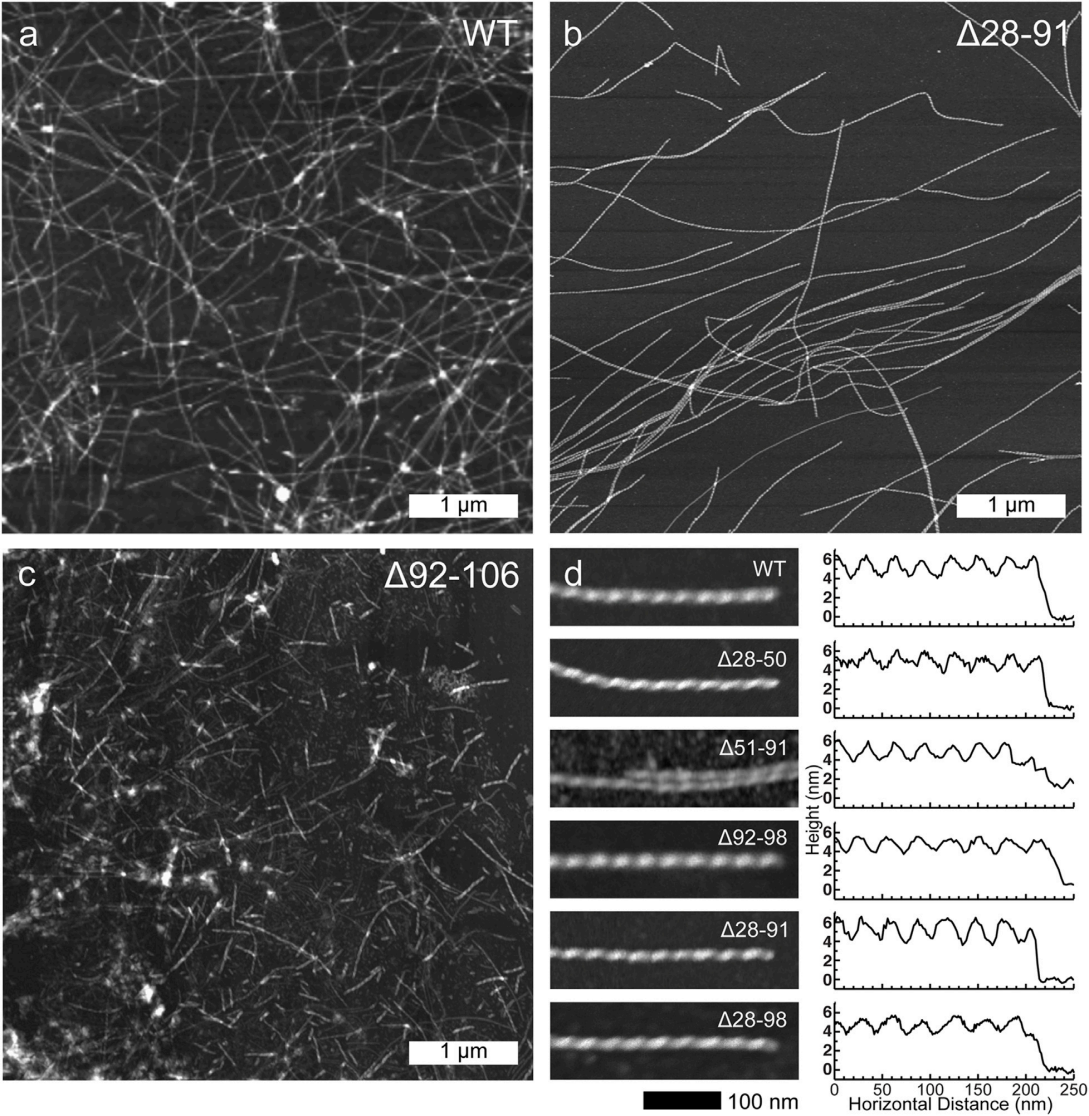
Representative low-resolution atomic force microscopy images for **(A)** WT, **(B)** Δ28-91 and **(C)** Δ92-106 huPrP23-144 fibrils (scale bar 1 μm for all panels). **(D)** High-resolution atomic force microscopy images (left; scale bar 100 nm for all panels) and corresponding height profiles (right) for WT, Δ28-50, Δ51-91, Δ92-98, Δ28-91, and Δ28-98 huPrP23-144 fibrils as indicated in the insets.

Atomic force microscopy was then used to investigate the morphologies of the resulting amyloid fibrils. With exception of the Δ92-106 fibrils, all the other deletion variants displayed morphologies that were similar to one another as well as to the morphology of WT huPrP23-144 amyloid. Specifically, as shown in Figure 2, these fibrils had highly uniform, micron long, threadlike morphologies with left-handed twist characterized by heights of ∼5–6 nm and periodicities of ∼30 nm. In contrast, the Δ92-106 fibril sample was far more heterogeneous, containing shorter fibrils of varying lengths in the range of tens to hundreds of nanometers as well as considerable amounts of apparently amorphous, non-fibrillar aggregates (Figure 2C).

**FIGURE 3:**
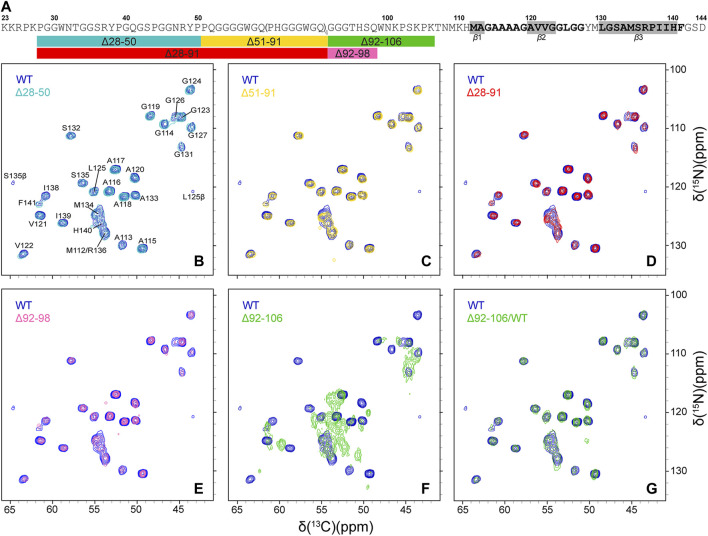
**(A)** Amino acid sequence of huPrP23-144. Relatively rigid residues comprising the amyloid core detected in conventional solid-state NMR experiments are shown in bold black font, with regions having highest *β*-strand propensity highlighted in grey rectangles (Theint et al., 2017b), and conformationally flexible residues (Helmus et al., 2008; Helmus et al., 2010) are shown in grey font. The rectangles below the amino acid sequence schematically show the key huPrP23-144 large deletion variants that were investigated in this study, with the colors of the rectangles corresponding to the colors of the contours in the solid-state NMR spectra in panels **(B)**-**(G) (B–G)** 2D ^15^N-^13^C*α* chemical shift correlation solid-state NMR spectra of Δ28-50 (b, cyan), Δ51-91 (c, gold), Δ28-91 (d, red), Δ92-98 (e, magenta), Δ92-106 (f, green) huPrP23-144 fibrils and Δ92-106 fibrils seeded with WT huPrP23-144 amyloid (g, green), overlaid with the reference spectrum for WT huPrP23-144 fibrils (blue).

**FIGURE 4:**
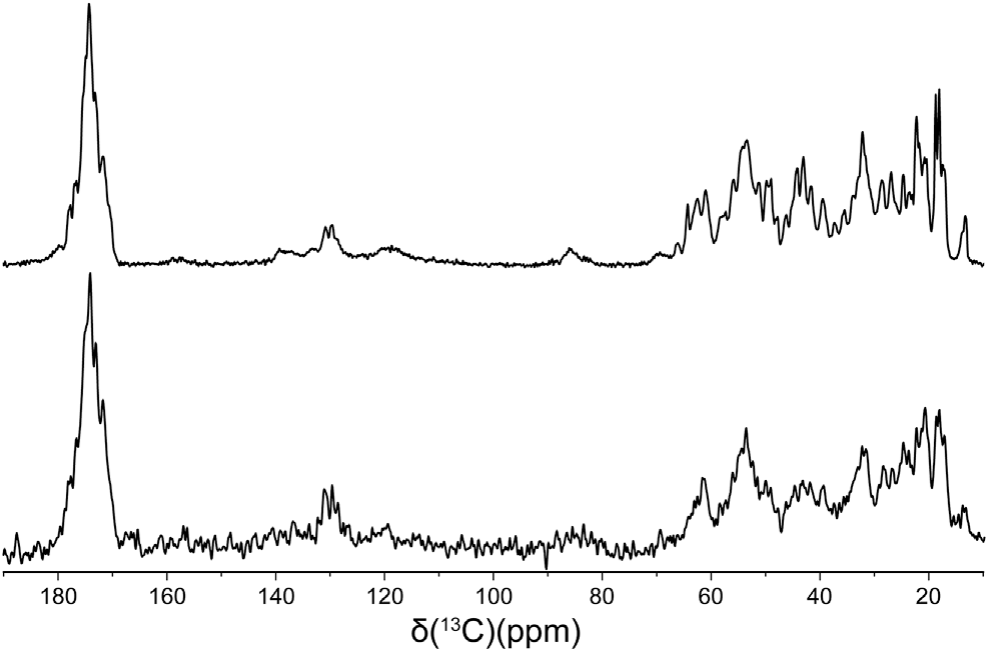
^13^C CP-MAS spectra of ^13^C,^15^N WT (top) and natural abundance Δ28-98 (bottom) huPrP23-144 amyloid fibrils.

Finally, in order to compare the protein conformations and degree of molecular ordering for the different fibril samples at the atomic level, we recorded two-dimensional (2D) fingerprint ^15^N-^13^Cα chemical shift correlation solid-state NMR spectra for wild-type huPrP23-144 amyloid and all the deletion variants except Δ28-98. Figure 3 shows the resulting 2D ^15^N-^13^Cα spectra, which were found to be effectively identical in terms of NMR signal frequencies, linewidths and relative intensities for WT huPrP23-144 and all deletion variants with exception of Δ92-106 fibrils; the latter exhibited considerable spectral differences relative to the other samples (Figure 3F). Note that for fibrils formed from the Δ28-98 variant, which did not express at a sufficiently high level in ^13^C and ^15^N isotope labeled minimal media to permit collection of a^15^N-^13^Cα correlation spectrum, a 1D ^13^C CP-MAS spectrum could be recorded for unlabeled fibrils (Figure 4). Apart from the obviously lower sensitivity associated with the use of a natural abundance sample, this spectrum showed considerable similarity to reference ^13^C CP-MAS spectrum recorded for ^13^C,^15^N-labeled WT huPrP23-144 fibrils. Altogether these data indicate that other than Δ92-106, all the huPrP23-144 deletion variants studied form amyloids that are highly ordered at the atomic level and possess WT-like core structures and core residue backbone motions. On the other hand, the spectra of Δ92-106 fibrils are indicative of structural perturbation/polymorphism for the core residues and a higher degree of molecular disorder, consistent with the higher degree of sample heterogeneity observed by AFM. Comparison of 2D ^15^N-^13^Cα spectra for WT and Δ92-106 fibrils reveals that they key chemical shift perturbations involve residues ∼112–115. This suggests that conformations of the β1-strand and several following core residues are primarily impacted in the deletion variant, in line with earlier studies (Theint et al., 2017a) indicating that several residues in this regime appear to be key for stabilizing the core fold of WT huPrP23-144 amyloid. Remarkably, however, we also find that seeding Δ92-106 amyloid formation with pre-formed WT huPrP23-144 fibrils results in the structural adaptation of the Δ92-106 protein to the WT huPrP23-144 amyloid core fold as evidenced by their indistinguishable 2D ^15^N-^13^Cα spectra (Figure 3G).

## Discussion

Collectively, on the basis of effectively identical fibril assembly kinetics, morphologies and fingerprint solid-state NMR spectra for all huPrP23-144 variants studied containing large deletions up to residue 98, our results indicate that the bulk of the dynamically disordered N-terminal tail domain of huPrP23-144 is not essential for amyloid formation under autocatalytic conditions and ability of the resulting *β*-core region to adopt a WT-like structure. Remarkably, this finding is strongly correlated with our previously reported data showing that proteinase-K resistant fragments of WT huPrP23-144 amyloid fibrils span residues 97–144, 98–144 and 99–144 (Jones et al., 2011).

Combined with the finding that the protein conformation and degree of molecular ordering appear to be significantly perturbed for the Δ92-106 deletion variant relative to WT huPrP23-144 and the different large huPrP23-144 deletion variants up to residue 98, our data indicate that aa ∼99–106 (and presumably several additional amino acids preceding the structured and relatively rigid fibril core beginning around residue Met-112) play a key role in stabilizing the formation of the characteristic huPrP23-144 *β*-core structure in spite of their flexible nature and location outside of the structured amyloid core region. Given that the 100–110 stretch of huPrP23-144 contains four positively charged lysine residues (at positions 101, 104, 106 and 110) we speculate that the stabilization of the amyloid core structure in WT huPrP23-144 occurs via electrostatic interactions with the negatively charged C-terminal aspartate residue.

While deletion of amino acids in the 99–106 regime clearly impacts the amyloid core structure and molecular ordering of huPrP23-144 within fibrils formed under autocatalytic conditions as discussed above, we also find that the seeding of fibril formation by the Δ92-106 deletion variant with pre-formed WT huPrP23-144 fibrils at low concentration leads to the Δ92-106 proteins adopting a WT-like core structure at the atomic level as revealed by the fingerprint ^15^N-^13^Cα NMR spectrum that is effectively identical to the corresponding spectrum for WT huPrP23-144 amyloid. Interestingly, this structural templating process that yields a WT-like fold for Δ92-106 amyloid was found to not appreciably alter the overall morphology of the Δ92-106 fibrils as viewed by AFM, with the majority of the sample consisting of relatively short fibrils similar to those observed in the unseeded reaction.

In summary, we systematically evaluated the influence of the dynamically disordered N-terminal tail domain of huPrP23-144 on the structure adopted by the amyloid core region by using deletion mutagenesis combined with magic-angle spinning solid-state NMR spectroscopy. We find that N-terminal huPrP23-144 residues up to aa 98, which coincide with the protein segment most susceptible to proteinase-K digestion in mature fibrils, can be deleted without impacting the core structure formed in an autocatalytic fibril assembly process. Remarkably, deletion of additional flexible residues (aa 99–106) located outside the amyloid core leads to the formation of amyloid fibrils with a perturbed core structure and reduced degree of molecular ordering in the fibril lattice, most likely caused by the disruption of stabilizing electrostatic interactions involving several lysine side-chains found in this region and the C-terminal aspartate residue. These structural perturbations, however, can be alleviated by catalyzing the amyloid formation with preformed WT huPrP23-144 fibril seeds.

## Materials and Methods

### Protein Expression and Purification

The plasmid encoding human PrP23-144 was described previously (Kundu et al., 2003) and plasmids encoding huPrP23-144 deletion variants were generated similarly to our previous study (Jones et al., 2011) by deletion mutagenesis using a QuikChange kit (Stratagene). Uniformly ^13^C,^15^N labeled huPrP23-144 and deletion variants were expressed in *E. coli* BL21 (DE3) cells and purified by nickel affinity chromatography as described in detail in previous studies (Theint et al., 2017b; Dao et al., 2021). The identities and purities of the resulting proteins were routinely confirmed by SDS/PAGE and MALDI mass spectrometry.

### Amyloid Fibril Formation

Amyloid fibrils were prepared under quiescent conditions at 25°C as described in previous studies (Theint et al., 2017a; Theint et al., 2017b; Dao et al., 2021). Lyophilized huPrP23-144 variants were dissolved in ultrapure water at ∼400 μM concentration and 1 M potassium phosphate buffer at pH 6.4 was added to a final concentration of 50 mM. For samples seeded with WT huPrP23-144 amyloid, 2% (mole fraction) of preformed fibrils was added immediately to the reaction after addition of phosphate buffer.

### Thioflavin T Fluorescence and Atomic Force Microscopy

Kinetics of fibril formation were monitored via the standard ThT fluorescence assay (Naiki et al., 1989) as described in detail in previous studies (Theint et al., 2018). Fibril morphologies were assessed by atomic force microscopy (AFM) as follows. Fibril suspensions were diluted 50-fold in ultrapure water, deposited on freshly cleaved mica substrates (Ted Pella Inc.) for 5 min, rinsed with three 50 μL aliquots of ultrapure water, and allowed to air dry for 1-2 h prior to imaging. Images were collected using a Bruker Dimension Icon AFM in PeakForce quantitative nanomechanical mapping mode with a ScanAsyst-Air probe and processed with the Bruker NanoScope Analysis software.

### Solid-State NMR Spectroscopy

Fibril suspensions for solid-state NMR analysis were incubated as described above for 48 h and centrifuged. The fibril pellets were washed three times with 50 mM potassium phosphate pH 6.4 buffer and packed into 3.2 mm zirconia rotors by centrifugation with the final samples containing ∼5–10 mg of fibrils. Standard 1D ^13^C CP-MAS and 2D ^15^N-^13^Cα solid-state NMR spectra were recorded on 500 MHz Varian and 800 MHz Bruker spectrometers, equipped with 3.2 mm triple resonance (^1^H-^13^C-^15^N) BioMAS and E^free^ probes, respectively. The sample temperature and MAS frequencies were actively regulated at 5°C and 11.111 kHz, respectively, and the experimental parameters were similar to those used in our previous solid-state NMR studies of WT huPrP23-144 fibrils (Helmus et al., 2008) with data acquisition times for the 2D ^15^N-^13^Cα NMR spectra of 8–16 h per sample. NMR spectra were processed and analyzed using NMRPipe (Delaglio et al., 1995) and Sparky (Goddard and Kneller, 2006), respectively.

## Data Availability

The original contributions presented in the study are included in the article/supplementary material further inquiries can be directed to the corresponding author.
